# Population pharmacokinetics and dose optimization of ceftazidime in critically ill children

**DOI:** 10.3389/fphar.2024.1470350

**Published:** 2024-11-27

**Authors:** Mengting Li, Liuliu Gao, Zuo Wang, Lingkong Zeng, Chen Chen, Jun Wang, Sichan Li, Maochang Liu, Yang Wang

**Affiliations:** ^1^ Department of Clinical Pharmacy, Wuhan Children’s Hospital (Wuhan Maternal and Child Healthcare Hospital), Tongji Medical College, Huazhong University of Science and Technology, Wuhan, China; ^2^ Department of Neonatology, Wuhan Children’s Hospital (Wuhan Maternal and Child Healthcare Hospital), Tongji Medical College, Huazhong University of Science and Technology, Wuhan, China; ^3^ Department of Pharmacy, Union Hospital, Tongji Medical College, Huazhong University of Science and Technology, Wuhan, China; ^4^ Drug Clinical Trial Institution, Wuhan Children’s Hospital (Wuhan Maternal and Child Healthcare Hospital), Tongji Medical College, Huazhong University of Science and Technology, Wuhan, China

**Keywords:** pediatric intensive care unit, children, ceftazidime, population pharmacokinetics, dose optimization

## Abstract

**Objective:**

The aim of this study was to develop a population pharmacokinetic model for ceftazidime in critically ill children in the pediatric intensive care unit (PICU) and optimize an appropriate dosing regimen for this population.

**Methods:**

We performed a prospective pharmacokinetic study on critically ill children aged 0.03–15 years. A population pharmacokinetic model was developed using the NLME program. Statistical and graphical methods were used to assess the stability and predictive performance of the model. Monte Carlo simulations were conducted to determine the optimal ceftazidime dosing regimen to achieve 70% *f*T > minimum inhibitory concentration (MIC).

**Results:**

This study included 88 critically ill children and 100 ceftazidime serum concentrations. The pharmacokinetic characteristics of ceftazidime were best described by a one-compartment linear elimination model. The weight and estimated glomerular filtration rate (eGFR) were determinant covariates for the clearance (CL) of ceftazidime. The recommended ceftazidime dosage regimens achieved a probability of target attainment (PTA) >90% for critically ill children at MIC values of 2, 4, and 8 mg/L. For bacterial infection at an MIC of 16 mg/L, it is difficult to achieve effective pharmacodynamic (PD) targets *in vivo* with the commonly used dose of ceftazidime.

**Conclusion:**

The population pharmacokinetic model of ceftazidime was established in critically ill children. Based on this model, we recommend evidence-based, individualized dosing regimens for subgroups with different weights and renal functions. The current daily dosage for children adequately meets the treatment requirements for MICs of 2, 4, and 8 mg/L, while for bacterial infection at an MIC of 16 mg/L, an elevated dosage regimen may be required.

**Clinical Trial Registration:**

https://www.medicalresearch.org.cn/login, Identifier MR-42-22-000220.

## 1 Introduction

Antibiotics are essential for prevention and treatment in the intensive care unit (ICU). The timely and adequate use of antibiotics can reduce mortality rates among critically ill patients (MacArthur et al., 2004).

Ceftazidime is the primary treatment for critically ill patients with *Pseudomonas aeruginosa* infections, commonly resulting from nosocomial pneumonia (ventilator-associated), catheter-associated urinary tract infections, or sepsis in the pediatric ICU (PICU) (Horino et al., 2012; Kang et al., 2003; Dantas et al., 2014). Ceftazidime has a low protein-binding value and is predominantly eliminated by the kidney (80%–90%), which may lead to its pharmacokinetics being particularly susceptible to changes in renal clearance (CL) (Cojutti et al., 2019). Ceftazidime has a half-life of 1.5–2.5 h in patients with normal renal function, which can extend to 15 h in individuals with impaired renal function (Ohkawa et al., 1985). Hemodynamic alterations may result in an increased renal CL in up to 65% of critically ill patients, with critically ill children potentially showing higher CL and greater volume of distribution (V_d_) than their healthy counterparts (Dhont et al., 2018; Van Der Heggen et al., 2019). In the PICU population, up to 95% of patients experience antibiotic concentrations outside the therapeutic range due to alterations in V_d_, protein binding, and CL (Tsai et al., 2015; Cies et al., 2018). This makes it challenging to achieve optimal drug concentrations that ensure efficacy while minimizing toxicity (Hartman et al., 2019).

For time-dependent antibiotics like ceftazidime, the pharmacodynamic (PD) target commonly focuses on the time that the free drug concentration remains above the minimum inhibitory concentration (*f*T > MIC). Animal infection models have indicated that a bacteriostatic effect can be achieved with a PD target of 40% *f*T > MIC, while 65% *f*T > MIC is necessary for maximum bactericidal activity within a 24-h period (Levison and Julie, 2009). For adults, an effective dosing regimen requires that drug concentrations remain above the MIC of pathogenic bacteria for a duration of at least 40%–50% of the dosing interval (Zhang et al., 2020). Given the escalating issue of antimicrobial resistance and the diverse nature of pathogens encountered in the PICU, our study adopted a more stringent PD target of 70% *f*T > MIC.

Few ceftazidime population pharmacokinetic (PPK) studies have been conducted in critically ill pediatric patients. Bui et al. (2020) established a PPK model for ceftazidime in critically ill children with cystic fibrosis and reported the significant impact of body weight and renal function on ceftazidime disposition, but the optimal dosage for critically ill children with varying renal functions has not been reported. Zhou et al. (2021) developed a physiologically based pharmacokinetic model for children with renal impairment and utilized it to evaluate pharmacodynamic parameters; however, it only offers a rough estimate of the dosage for pediatric patients with renal impairment.

In the present study, we identified factors that influence ceftazidime CL at the first step and then established a PPK model specific to critically ill children. Additionally, we aimed to evaluate the ceftazidime dosing regimen currently used and recommend dosing regimens that achieve optimal PD target attainment (70% *f*T > MIC).

## 2 Materials and methods

### 2.1 Study population

A prospective, open-label PPK study on ceftazidime was carried out in the PICU at Wuhan Children’s Hospital, Tongji Medical College, Huazhong University of Science and Technology, between October 2019 and January 2020. The patients were diagnosed with or suspected of having a bacterial infection and received intravenous ceftazidime treatment. Eligibility criteria were (1) age 18 years or younger and (2) undergoing ceftazidime treatment for a minimum of 3 consecutive days. Patients were excluded if they were involved in other clinical trials, had incomplete dose information, or were intolerant to ceftazidime treatment.

The study adhered to the Declaration of Helsinki and relevant legal regulations and was approved by the Wuhan Children’s Hospital Ethics Committee (approval number 2021R153-E03). All patients or their legal representatives provided written informed consent. This study is registered in the China National Medical Research Registration and Filing Information System under clinical trial number MR-42-22-000220, https://www.medicalresearch.org.cn/login.

### 2.2 Dosing regimen and sample collection

Ceftazidime was administered intravenously at 25–100 mg/kg, with a dosage tailored to the patient’s clinical status. Blood samples were collected through opportunistic blood sampling. For each patient, 1–3 samples were obtained, with a volume of 2 mL drawn per sample for analysis. The blood samples were immediately centrifuged for 10 min to separate the serum, which was used for ceftazidime concentration analysis. The administration and sampling times were precisely documented.

Demographic and laboratory parameters recorded included gender, age, height, weight, serum creatinine concentration (SCR), blood urea nitrogen (BUN), serum cystatin C (Cys-C), uric acid (UA), alanine aminotransferase (ALT), aspartate aminotransferase (AST), and total bilirubin (TBIL). The serum creatinine level was measured using the enzymatic method using Roche cobas 8000 c702, as reported in previous studies. The estimated glomerular filtration rate (eGFR) was obtained by using the modified Schwartz formula [eGFR (mL/min·1.73 m^2^) = 0.413*(height/serum creatinine)] (Schwartz et al., 2009).

### 2.3 Analytical methods

Ceftazidime was extracted from the serum using a Cleanert ODS C18 SPE Column (200 mg/3 mL). A 0.5 mL serum sample was added to the column (Cleanert ODS C18, Agela Technologies) and eluted with 50% acetonitrile. The elution was examined using a validated HPLC system with ultraviolet (UV) detection (1260 Infinity II; Agilent Technologies Inc., Santa Clara, CA) at 30°C. UV detection was conducted at a wavelength of 230 nm. The mobile phase was a mixture of sodium dihydrogen phosphate and acetonitrile (75:25, pH 3.3) delivered at a flow rate of 0.8 mL/min. Ceftazidime in serum exhibited a linear range of 0.025–100 μg/mL, with a detection limit of 0.025 μg/mL. The precision for both intra- and inter-day measurements was below 10%.

### 2.4 PPK model building

The PPK model was performed using Phoenix^®^ NLME software (version 8.2, Pharsight Corporation, Mountain View, CA, United States). R software (version 3.5.1, https://www.r-project.org/) was used for statistical tests and graph generation.

The PPK model comprises structural models and random-effects models. Structural models describe the concentration–time relationship, and random-effects models assess intra- and inter-individual variability. Both one- and two-compartment structural models with linear or nonlinear elimination kinetics were constructed to fit the ceftazidime concentration–time data. The structural model was assessed using variations in the objective function value (OFV) and visual diagnostic plots.

Inter-individual variability (η_i_) was best described by the exponential model as Equation 1:

$$\text{Pi}=\theta *\exp\left(\eta_{i}\right).$$ (1)

Here, Pi denotes the estimated pharmacokinetic parameter for individual i, θ represents the typical population parameter value, and η_i_ indicates inter-individual variability, modeled as a normal distribution with a mean of 0 and variance ω^2^.

The intra-individual variability, also known as residual variability, was characterized using an additive model (Equation 2), a proportional model (Equation 3), and a combined additive model (Equation 4) respectively. The equations are as follows:

Y=IPRED+ε, (2)

$$Y=\text{IPRED}\times \exp\left(1+\varepsilon \right),$$ (3)

$$Y=\text{IPRED}\times \exp\left(1+\varepsilon_{1}\right)+\varepsilon_{2}.$$ (4)

Here, Y denotes the observation, IPRED is the individual prediction, and ε_n_ signifies the residual error of the model, characterized by a mean of 0 and a variance of σ^2^.

### 2.5 Covariate analyses

Potential covariates for the pharmacokinetic parameters included demographic characteristics (gender, age, weight, height, and body surface area [BSA]), renal functions (SCR, BUN, cystatin C [Cys-C], and uric acid [UA]), and hepatic functions (ALT, AST, TBIL, and eGFR). A preliminary evaluation of the correlation between covariates and ceftazidime CL was conducted using hypothesis testing. Covariates with *p*-values below 0.05 were included in the model. Covariates were selected using a previously described stepwise method (Mould and Upton, 2013). In forward selection, each covariate was added individually to the base model. A covariate was deemed significant if it resulted in a reduction in the OFV of >3.84 (*p* < 0.05). Subsequently, the basic model was expanded into a full model by integrating all covariates. In the backward elimination, a covariate with an increase in the OFV of < 6.635 (*p* < 0.01) was removed from the full model. Considering that the maturation and development of children greatly impact CL, five different maturation models based on allometric scaling were applied to optimize the full model and establish the final model.

### 2.6 Model validation

The final model was assessed by the inspection of the goodness-of-fit (GOF) plots for diagnostic evaluation. The GOF plots included observed concentrations versus individual prediction (DV vs. IPRED), DV versus population prediction (PRED), conditional weighted residuals (CWRES) versus PRED, and CWRES versus time after dose. The stability and performance of the final model were verified by the nonparametric bootstrap method, which involved resampling 1,000 replicated datasets from random sampling. The estimated median values from the 95% confidence intervals (95% CIs) in the bootstrapping step were compared to those from the final model. The fit of the final model to the data was further assessed using the normalized prediction distribution error (NPDE), with the results displayed graphically through quantile–quantile plots, NPDE histograms, NPDE versus PRED plots, and NPDE versus time-after-dose plots.

### 2.7 Dosing regimen evaluation and optimization

The PD target of ceftazidime in the study was set at 70% of the time the free drug concentration remains above the minimum inhibitory concentration (70% *f*T > MIC) during the dosing interval. To achieve an optimal balance between maximum bactericidal efficacy and minimal venous catheter occupancy while ensuring safety during resuscitation, the criteria for dose optimization were as follows: 1) the dosage should ensure that greater than 90% of the subgroup patients could reach the PD target (70% *f*T > MIC) during the therapy, that is, PTA >90% (Europe Medicine Agency, 2016) and 2) the administration frequency was set at 2–4 times per day. Within the dosing range of 25–100 mg/kg/day, a less frequent regimen was prioritized over a lower dose amount, while within the dose >100 mg/kg/day, lower-dose regimens represent the optimized approach. The free concentration of ceftazidime was determined by 90% of the total plasma concentration (Li et al., 2021). Based on the estimated parameters from the final model, simulations were conducted for various dosing regimens in decreasing order of dose levels. The Monte Carlo simulation (n = 1,000) was conducted to predict whether ceftazidime can achieve the therapeutic target of PTA >90%. MIC values of 2, 4, 8, and 16 mg/L were used to calculate the PTA in Monte Carlo simulations.

## 3 Results

### 3.1 Study population

The study included 100 ceftazidime serum concentrations collected from 88 patients. Of these participants, 63.6% were male and 36.4% were female. No patients discontinued the ceftazidime treatment due to adverse events. Patient ages ranged from 0.03 to 15 years, with a mean age of 5.43 ± 4.1 (SD) years. The patients were administered intravenous ceftazidime with a median loading dose of 48.38 mg/kg, ranging from 21.05 to 76 mg/kg. Serum concentrations in the cohort varied from 0.046 mg/L to 74.84 mg/L, with a median trough level of 3.35 mg/L. The study categorized the children based on renal function as follows: moderate insufficiency (eGFR 30–60 mL/min·1.73 m^2^) with 5 children; mild insufficiency (eGFR 60–90 mL/min·1.73 m^2^) with 9 children; normal function (eGFR 90–120 mL/min·1.73 m^2^) with 31 children; and augmented function (eGFR 120–200 mL/min·1.73 m^2^) with 43 children. Further physiological characteristics and demographic data are given in Table 1.

**TABLE 1:** Demographic and physiological characteristics of the patients.

Parameter	Number	Mean (SD)	Median (range)
Patients	88		
Gender	56M/32 F		
Samples	100		
Age (years)		5.43 (4.10)	5.17 (0.03–15)
Weight (kg)		21.07 (14.70)	18.35 (2.80–95)
Height (cm)		108.33 (32.45)	111 (50–172)
BSA (m^2^)		0.78 (0.37)	0.76 (0.20–2.09)
BUN (mmol/L)		4.69 (2.51)	4.19 (1–14.1)
SCR (μmol/L)		34.99 (12.10)	33.9 (15.9–66.2)
UA (μmol/L)		317.11 (160.70)	265.3 (38–789)
TBIL (μmol/L)		22.04 (52.55)	10.1 (2.9–408.7)
ALT (U/L)		62.45 (220.96)	20.5 (4–1,672)
AST (U/L)		141.11 (785.87)	29 (9–7,758)
eGFR (ml/min·1.73 m^2^)		116.41 (31.35)	116.93 (40.42–197.15)
Ceftazidime serum concentration (µg/mL)		8.82 (15.67)	3.35 (0.046–74.84)

The eGFR was calculated using the modified Schwartz formula: eGFR (ml/min·1.73 m^2^) = 0.413*(Height/Serum creatinine). The BSA was calculated using the Mosteller formula: BSA (m^2^) = {(height [cm] * weight [kg])/3,600}^1/2^.

### 3.2 PPK model building

The pharmacokinetic properties of ceftazidime were effectively described by a one-compartment model with linear elimination. Although the two-compartment model showed a 4.63% reduction in the OFV compared to the one-compartment model, its PK parameter estimation was inadequate due to data distribution issues from opportunistic sampling. Furthermore, the OFV for the nonlinear elimination model was greater than that for the linear model, and it did not align with the CL characteristics of ceftazidime *in vivo*. Considering these findings and prior studies, a one-compartment linear elimination model was chosen as the foundational structural model, parameterized by V_d_ and CL.

**FIGURE 1:**
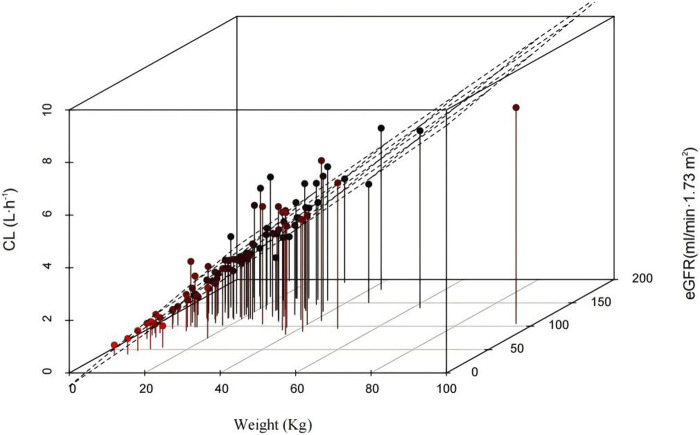
3D scatter plot of the relationship for the CL of ceftazidime between weight and eGFR.

**FIGURE 2:**
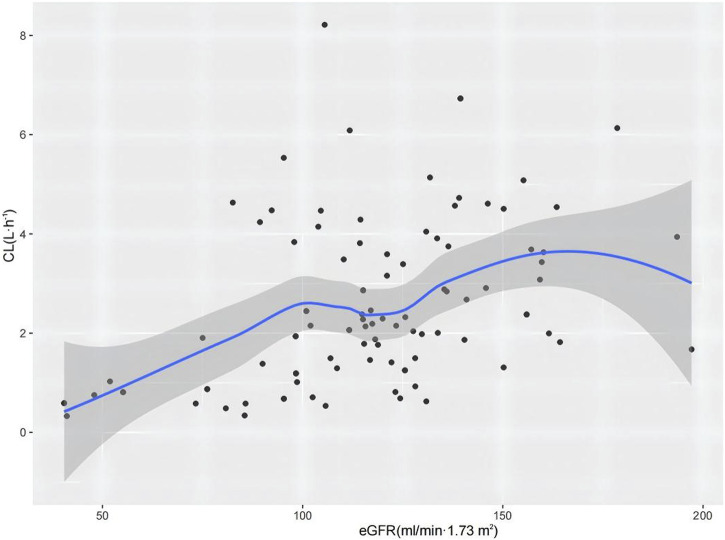
CL of ceftazidime versus eGFR profile. The shaded area represents the 95% confidence intervals for the locally weighted regression.

The results of the hypothesis test are given in Supplementary Table S1. The details of the covariate screening process by a stepwise approach are given in Table 2 (steps 1–4). In the forward-selection process, age, weight, height, BSA, BMI, Cys-C, and eGFR were incorporated into the basic structural model. Weight was considered a fixed covariate, reducing the OFV by 17.49 points. Additionally, the eGFR significantly affected ceftazidime CL, decreasing the OFV by 15.87 points. In the backward elimination, no covariate was removed from the full regression model, and both weight and eGFR were retained in the model as determinant variables for CL. Figure 1 shows a 3D scatter plot with vertical lines and a regression plane that was used to visualize the CL of ceftazidime. The plot indicated that CL increased with both body weight and eGFR. As shown in Figure 2, the relationship between the eGFR and CL was visualized by the scatterplot smoothing method. The result indicated a positive correlation between ceftazidime CL and eGFR.

**TABLE 2:** Final model development process and statistical analysis.

Step	Covariates screening	OFV	△OFV	*p*-value	Comments
0	None	630.77			Base model
	Forward selection				
1	CL-WT	613.28	−17.49	<0.05	
2	CL-WT/V_d_-WT	567.43	−45.85	<0.05	
3	CL-WT-eGFR/V_d_-WT	551.56	−15.87	<0.05	Full model
	Backward elimination				
4	No covariate excluded	—	—	—	
	Maturation model optimization				
5	Model I: The 3/4 allometric model	559.31	—	—	
6	Model II: The simple exponent model	551.56	—	—	Final model
7	Model III: The maturation model	552. 28	—	—	
8	Model IV: The weight-dependent exponent model	550.73	—	—	Parameters were highly variable
9	Model V: The age-dependent exponent model	551.16	—	—	Model structure was unstable

The optimization process of maturation models is given in Table 2 (steps 5–9). Models I and III were excluded due to their higher OFVs. Although models IV and V had lower OFVs than model II, their parameters were highly variable, and the structure was unstable. Therefore, model II showed the best fit and was identified as the final model. The equations to derive the population values of the final model for V_d_ and CL are shown in Equations 5, 6:

$$CL\left(L\cdot h^{-1}\right)=7.76\times {\left(\frac{weight}{70}\right)}^{0.9}\times {\left(\frac{eGFR}{116.93}\right)}^{0.38}\times \exp\left(\eta_{CL}\right),$$ (5)

$$V_{d}\left(L\right)=27.83\times \left(\frac{\text{weight}}{70}\right)\times \exp\left(\eta_{V_{d}}\right),$$ (6)

where the weight is given in kg and the eGFR is given in mL/min·1.73 m^2^. Table 3 presents the pharmacokinetic parameters and bootstrap results of the final model. The typical values of V_d_ and CL were 27.83 L and 7.76 L h^−1^, respectively, which were normalized by a weight of 70 kg and a median eGFR of 116.93 mL/min·1.73 m^2^.

**TABLE 3:** Parameter estimates and bootstrap results of the final model.

Parameter	Final model		Bootstrap analysis		Bias (%)
	Estimate	RSE (%)	Median estimate	95% CIs	
V_d_ (L)	27.83	7.85	27.96	21.04–37.70	0.47
CL (L·h^−1^)	7.76	10.40	7.79	6.14–10.03	0.39
*θ*_*1*_	1 (fixed)	—	1 (fixed)	—	—
*θ*_*2*_	0.90	6.41	0.91	0.81–10.03	1.11
*θ*_*3*_	0.38	33.59	0.36	0.13–0.60	−5.26
Inter-individual variability					
ω_Vd_^2^	0.04	39.77	0.04	0.002–0.07	0
ω_CL_^2^	0.06	21.40	0.06	0.03–0.09	0
Residual variability					
σ (mg·L^−1^)	1.20	14.60	1.17	0.67–1.52	−2.50

θ_1_, exponent for weight as a covariate for V; θ_2_, exponent for weight as a covariate for CL; θ_3_, exponent for the eGFR as a covariate for CL; ω_Vd_, square root of inter-individual variance for Vd; ω_CL_, square root of inter-individual variance for CL; σ, residual variability for the additive error.

Inter- and intra-individual variability were estimated for CL and V_d_. The additive model exhibited superior accuracy in predicting individual values compared to the proportional model and combined additive model. The bootstrap analysis indicated a stable and credible result since the median values of the bootstrap replicates (relative error <10%) were close to the estimated parameters of the final model, demonstrating its stability.

### 3.3 Model validation

**FIGURE 3:**
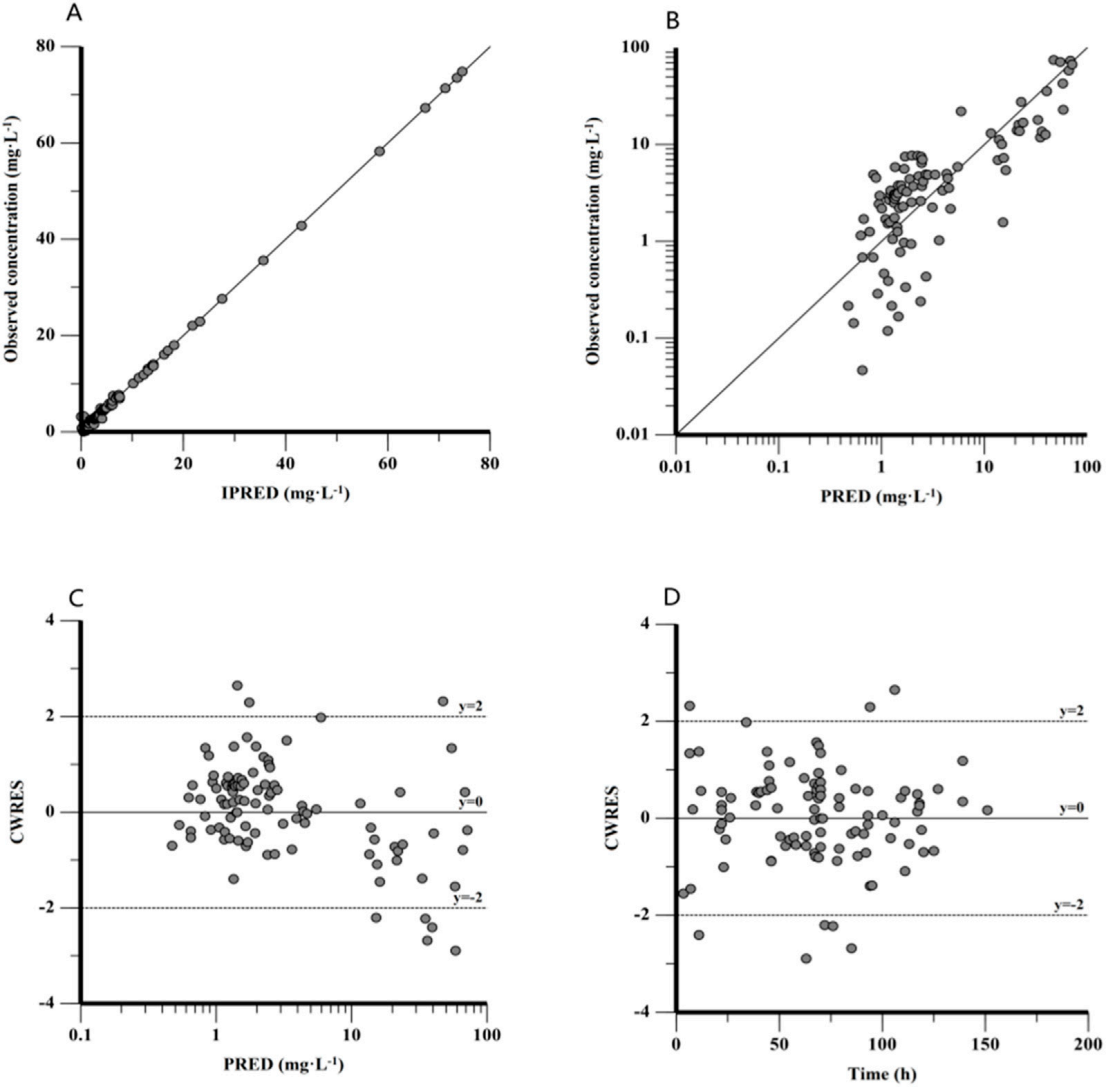
Goodness-of-fit plot for the final PPK model. **(A)** Observed concentration against individual predictions (IPRED), **(B)** observed concentration against population predictions (PRED), **(C)** conditional weighted residuals (CWRES) against PRED, and **(D)** CWRES against time after dose.

Figure 3 (Figures 3A–D) shows the GOF plots for the final model. Figures 3A, B show that both IPRED and PRED closely correspond to the observed concentrations, indicating a high prediction accuracy of the final model. Figures 3C, D show that the majority of CWRES fall within the ±2 range, indicating a satisfactory fit for the final model. The bootstrap method results showed that the median parameter estimates align with the final model predictions, with each parameter’s median value falling within the 5%–95% range without notable deviation.

**FIGURE 4:**
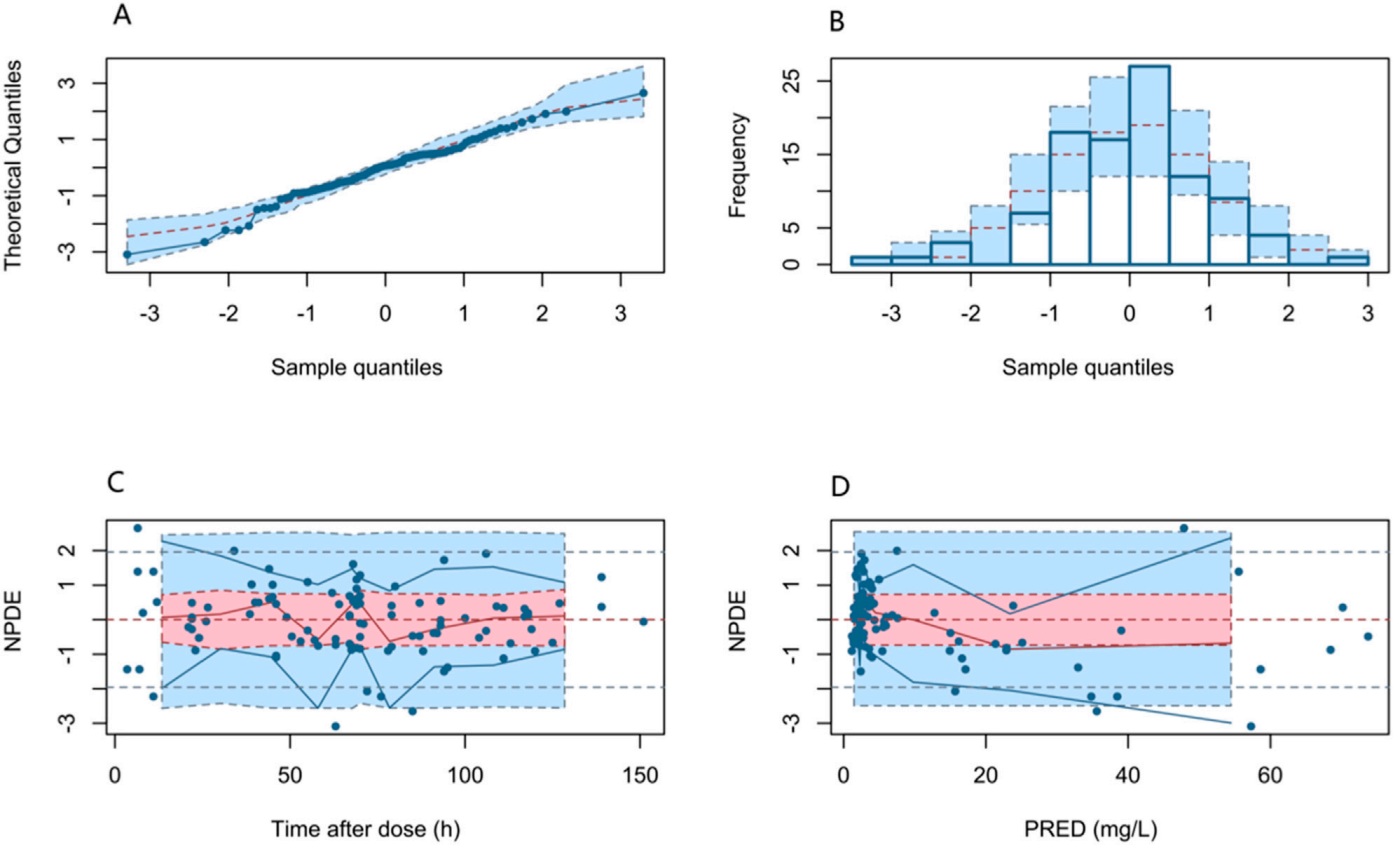
NPDEs of the final PPK model. **(A)** Quantile–quantile plot versus the expected standard normal distribution; **(B)** histogram of the NPDE with the density of the standard normal distribution overlaid; **(C)** scatter plot of the NPDE against time after dose; and **(D)** scatter plot of NPDE versus PRED.

Figure 4 shows the fit of the final model to the data assessed by the NPDE, yielding a mean of 0.026 (SE = 0.099) and a variance of 0.9788 (SE = 0.140). The NPDE results were evaluated with a t-test (*p* = 0.79), Shapiro–Wilks test (*p* = 0.24), Fisher’s variance test (*p* = 0.918), and a global test (*p* = 0.721). The statistical analysis indicated that the NPDE conformed to a theoretical N (0,1) distribution with variance homogeneity. Overall, the final PPK model presented good stability and predictive capability for PPK parameters.

### 3.4 Dosing regimen optimization

As noted, the weight and eGFR were the determinant variables for CL. Children were categorized into several subgroups based on body weight—(i) weight <10 kg, (ii) weight of 10–30 kg, (iii) weight of 30–50 kg, and (iv) weight of 50–70 kg—and eGFR—(i) moderate renal insufficiency, (ii) mild renal insufficiency, (iii) normal renal function, and (iv) augmented renal function. Supplementary Table S2 shows the PTA calculated based on the PPK model at an MIC of 2, 4, 8, and 16 mg/L for different ceftazidime dosage regimens. Table 4 shows the optimal ceftazidime dosages for various eGFR and weight subgroups. The results of the Monte Carlo simulations indicated that, all regimens can achieve a PTA >90% at an MIC of 2, 4, and 8 mg/L in critically ill children populations, whereas the commonly used dosage (25 mg–100 mg/kg, twice or thrice daily for children) of ceftazidime makes it difficult to achieve a PTA >90% for bacterial infection at an MIC of 16 mg/L, indicating that an elevated dosage is required.

**TABLE 4:** Recommended dosage of ceftazidime for children based on the simulation results of this study.

Patient group		Recommended dosage			
		Infection bacterial resistance			
Weight (kg)	eGFR (mL/min·1.73 m^2^)	MIC_90_ = 2 mg/L	MIC_90_ = 4 mg/L	MIC_90_ = 8 mg/L	MIC_90_ = 16 mg/L
<10	30–60	17.5 mg/kg q12 h	35 mg/kg q12 h	22.5 mg/kg q8 h	25 mg/kg q6 h
	60–90	32.5 mg/kg q12 h	17.5 mg/kg q8 h	32.5 mg/kg q8 h	35 mg/kg q6 h
	90–120	50 mg/kg q12 h	25 mg/kg q8 h	22.5 mg/kg q6 h	—
	120–200	20 mg/kg q8 h	16 mg/kg q6 h	30 mg/kg q6 h	—
10–30	30–60	12.5 mg/kg q12 h	22.5 mg/kg q12 h	50 mg/kg q12 h	32.5 mg/kg q8 h
	60–90	20 mg/kg q12 h	40 mg/kg q12 h	25 mg/kg q8 h	27.5 mg/kg q6 h
	90–120	30 mg/kg q12 h	17.5 mg/kg q8 h	32.5 mg/kg q8 h	32.5 mg/kg q6 h
	120–200	12.5 mg/kg q8 h	25 mg/kg q8h	22.5 mg/kg q6 h	—
30–50	30–60	10 mg/kg q12 h	20 mg/kg q12 h	40 mg/kg q12 h	30 mg/kg q8 h
	60–90	17.5 mg/kg q12 h	35 mg/kg q12 h	22.5 mg/kg q8 h	25 mg/kg q6 h
	90–120	25 mg/k q12 h	47.5 mg/kg q12 h	30 mg/kg q8h	30 mg/kg q6h
	120–200	42.5 mg/kg q12 h	20 mg/kg q8 h	20 mg/kg q6 h	40 mg/kg q6 h
50–70	30–60	10 mg/kg q12 h	17.5 mg/kg q12 h	35 mg/kg q12 h	27.5 mg/kg q8 h
	60–90	15 mg/kg q12 h	30 mg/kg q12 h	20 mg/kg q8 h	22.5 mg/kg q6 h
	90–120	22.5 mg/kg q12 h	45 mg/kg q12 h	25 mg/kg q8 h	27.5 mg/kg q6 h
	120–200	40 mg/kg q12 h	20 mg/kg q8 h	20 mg/kg q6 h	37.5 mg/kg q6 h

**FIGURE 5:**
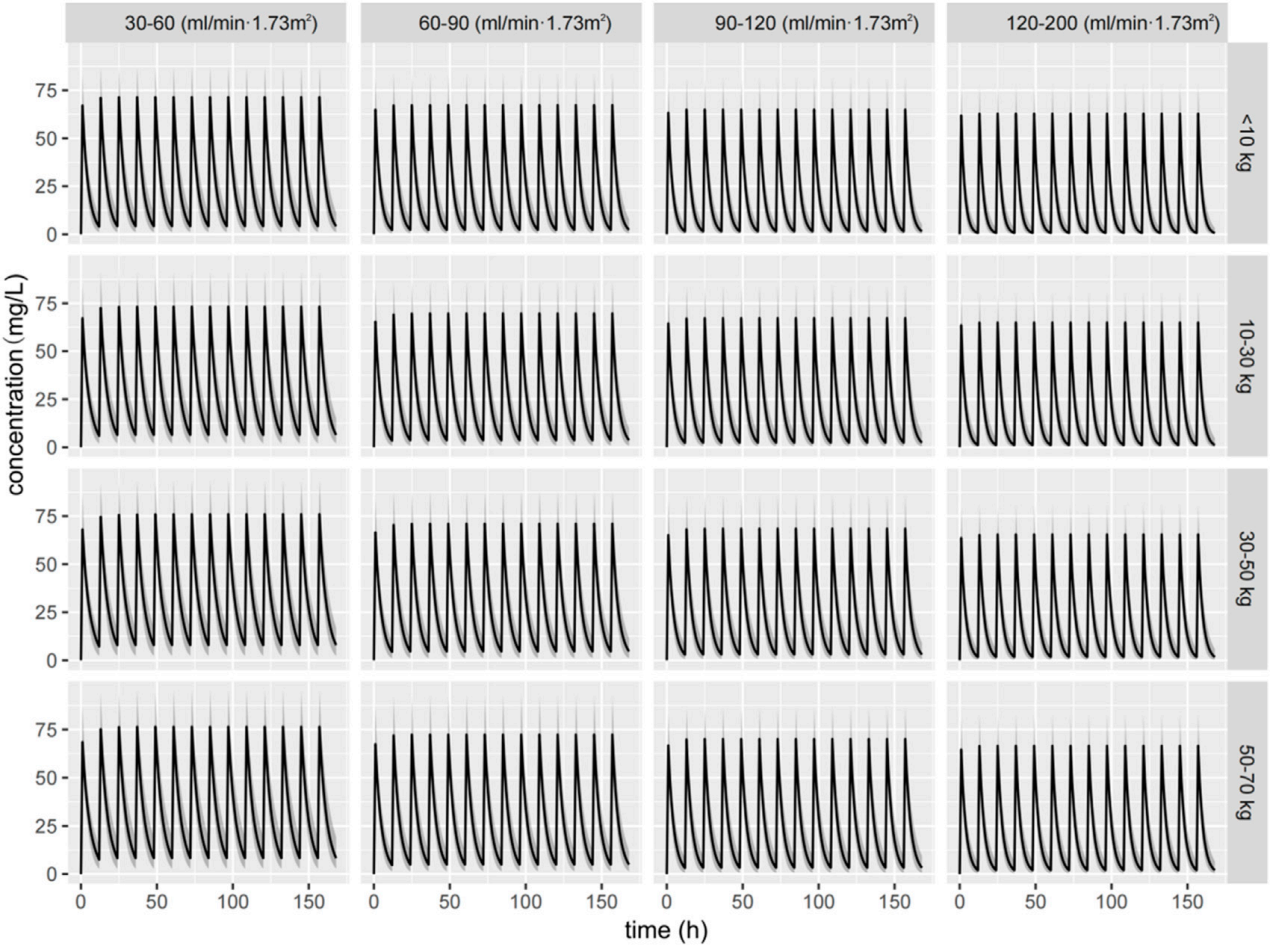
Simulated concentration–time curve of ceftazidime for children with different weights and eGFRs receiving a dose of 30 mg/kg q12 h dose.

Figure 5 graphically shows the impact of weight and renal function on the CL of ceftazidime. All patient subgroups were simulated to receive a standardized dose of 30 mg/kg every 12 h. In participants within the same-weight subgroup, the ceftazidime CL decreased, and the steady-state concentration increased with renal insufficiency, whereas CL increased and ceftazidime concentration decreased in cases with augmented renal function. Among patients with similar eGFRs, ceftazidime concentrations were lower for those weighing less than 10 kg, likely due to immature renal function in children.

## 4 Discussion

Previous studies have primarily focused on the PPK characteristics of ceftazidime in neonates, infants, and children. However, research regarding the dosage regimens for critically ill pediatric patients is still limited. Current PPK studies on ceftazidime often lack a comprehensive consideration of covariates such as age, weight, and renal function, potentially leading to less effective dosing regimen optimization.

Our study conducted a PPK analysis of ceftazidime involving 88 critically ill Chinese pediatric patients. Using a quantitative pharmacological approach, we successfully developed a PPK model, specifically tailored for critically ill children. A validated one-compartment linear elimination model was developed for ceftazidime. Both weight and eGFR were identified as significant covariates affecting the PPK of ceftazidime. Based on the simulations of dosage regimens, we proposed recommended dosages for ceftazidime treatment. These recommendations provide a direct and practical framework for adjusting ceftazidime dosages in pediatric patients based on their body weight and renal function.

The pharmacokinetics of hydrophilic antibiotics, such as ceftazidime, which are primarily cleared renally, can be significantly affected by alterations in renal function, edema, and resuscitation therapy. These factors may result in changes in both CL and V_d_ (Buning et al., 2021). Our study identified the eGFR as a significant covariate influencing ceftazidime CL, aligning with prior PPK research findings. Nguyen et al. (2021) demonstrated that renal function significantly affects ceftazidime elimination, with an OFV reduction of 26.26. Cojutti et al. (2019) conducted a pharmacokinetic analysis of continuous-infusion ceftazidime in febrile neutropenic children, identifying both renal function and body surface area as significant covariates of ceftazidime CL, with a median estimated CL of 3.18 L/h. Bui et al. (2020) reported that the CL of ceftazidime was 0.873 L/h in children without cystic fibrosis and 1.589 L/h in those with cystic fibrosis. In our study, the median estimated CL (7.76 L/h) in critically ill children was higher than the values previously observed. This variation is expected, given the distribution of children with renal function levels: 5 with moderate insufficiency, 9 with mild insufficiency, 31 with normal function, and 43 with augmented function. Nearly half of the cohort exhibited augmented renal clearance (ARC), resulting in a significantly elevated CL. There have been several reports regarding ARC in severely ill children. Lee et al. (2017) reported that 75% of critically ill children exhibited sub-therapeutic vancomycin trough levels due to ARC of vancomycin in this population. Dhont et al. (2018) reviewed the knowledge on ARC in critically ill children, indicating that many of these patients may experience ARC likely due to a hyperdynamic cardiovascular state caused by systemic inflammation, fluid resuscitation, and inotropic support. Furthermore, specific disease states, such as comorbidities, hematologic malignancies, trauma, or head injury, induce ARC for other hydrophilic antibiotics, as shown in previous reports (Akers et al. (2014); Drusano, 2004).

The selection of the MIC for pathogenic bacteria should be specific to the type of bacterial infection and the target patient population. Coagulase-negative *Staphylococci* and *Streptococcus pneumonia* had an MIC of 16 mg/L (Zhang et al., 2020), *P. aeruginosa* and *Staphylococcus aureus* had an MIC of 8 mg/L (Lubasch et al., 2003), while *Enterobacter* species (*Escherichia coli* and *Klebsiella pneumonia*) and Group B *Streptococcus* had an MIC of 4 mg/L (Khan et al., 2015). Other pathogenic bacteria had MIC values less than 4 mg/L (Goldstein and Citron, 1985). In our study, Monte Carlo simulations were conducted to optimize treatment for various bacterial infections at MIC values of 2, 4, 8, and 16 μg/mL.

Inadequate antibiotic dosing significantly increases the risk of developing drug resistance (Bui et al., 2020). Considering the notably high CL of ceftazidime in critically ill pediatric patients, it is essential to adjust its dosage accordingly. Existing guidelines provide a wide range of recommended dosages for ceftazidime in different pediatric age groups. For example, the domestic manual suggests a dosage of 25–60 mg/kg, administered twice daily for infants aged from newborns to 2 months, and 30–100 mg/kg, twice or thrice daily for children over 2 months old. The World Health Organization Model Formulary for Children (WMFC) advises a dosage of 25–50 mg/kg for treating infections from Gram-positive and Gram-negative bacteria, with the dosage frequency tailored to the age of neonates and children aged 1 month–18 years (Model Formulary for Children, 2010). In the British National Formulary for Children (BNFC), the recommended dosage for infections caused by Gram-positive and Gram-negative bacteria is 25 mg/kg, aligning with the WMFC guidelines. This dosage may be increased to 50 mg/kg in patients experiencing severe infections, meningitis, or febrile neutropenia. For pediatric patients aged 1 month–18 years with cystic fibrosis infected by *P. aeruginosa*, the recommended dosage is 50 mg/kg three times daily (British National Formulary for Children, 2015). In our research, we performed 1,000 Monte Carlo simulations to assess the efficacy of various pre-set doses. As shown in Table 4, for the bacterial infection at MICs of 2, 4, and 8 mg/L, the recommended dosage could achieve a PTA >90%. Under certain conditions at an MIC of 8 mg/L, more frequent administration may be required. Additionally, for children under 10 kg with an eGFR of 120–200 mL/min·1.73 m^2^, a dosage adjustment to 120 mg/kg may be warranted. For children weighing 30–70 kg with an eGFR of 30–60 mL/min/1.73 m^2^, a dose of merely 10 mg/kg every 12 h can achieve a PTA >90% at an MIC of 2 mg/L. Overall, the existing daily dosage for children, 25–100 mg/kg, administered twice or thrice daily, can adequately meet the treatment requirements for MICs of 2, 4, and 8 mg/L. For a bacterial infection at an MIC of 16 mg/L, no dosing regimen can achieve a PTA >90% for individuals under 10 kg with an eGFR of 90–200 mL/min/1.73 m^2^ or those weighing 10–30 kg with an eGFR of 120–200 mL/min/1.73 m^2^. In particular, for individuals weighing 30–50 kg with an eGFR of 120–200 mL/min/1.73 m^2^, a dosage of 160 mg/kg per day should be considered. In the domestic manual, higher doses (150 mg/kg per day, or a maximum of 6 g per day) are only suitable for immunocompromised children with cystic fibrosis or children with meningitis. However, high doses of β-lactam antibiotics are linked to an increased risk of renal failure and neurotoxicity (Imani et al., 2017). As β-lactam antibiotic PK/PD relationships are best described by ƒT > MIC, higher concentrations are unlikely to be of additional therapeutic effect (Leegwater et al., 2023). Thus, clinicians should carefully select the optimal ceftazidime dose, ensuring a balance between toxicity and antimicrobial efficacy for bacterial infections at an MIC of 16 mg/L. In addition to increasing the dosage or reducing the dosing interval, prolonging the infusion duration represents a viable consideration. For time-dependent antibacterial drugs, extending the infusion duration can enhance the proportion of time that the drug concentration remains above the MIC, thus boosting therapeutic effectiveness (Karaba et al., 2024). Bulitta et al. (2010) conducted Monte Carlo simulations to evaluate five ceftazidime dosing regimens, finding that both extended and continuous infusions achieved a PK/PD breakpoint of 8–12 mg/L in cystic fibrosis patients, approximately 10-fold higher than the breakpoint for the same daily dose administered via q8 h short-term infusion.

This study acknowledges several limitations. First, we did not consider the influence of other factors on ceftazidime PPK. In the PICU, children often present with multiple health issues, such as sepsis, cardiovascular problems, and other complications, which require various treatments. These include fluid resuscitation, concurrent therapies, mechanical ventilation, and nutritional support, all of which may affect the pharmacokinetic profile of ceftazidime. For example, in a previous PPK study, mechanical ventilation was identified as a significant covariate that resulted in a 2.5-fold increase in the volume of the peripheral compartment for ceftazidime (Conil et al., 2007). Therefore, further research is needed to explore the impact of complex covariates in greater detail. Second, the sample size of the research was not sufficiently large to provide a comprehensive representation of the entire population. This limitation is particularly evident when considering the subgroup of children with moderate and mild renal insufficiency. Future studies should aim to recruit a larger and more diverse sample size, ensuring that children with moderate and mild renal insufficiency are adequately represented. Despite the limitations, the study can provide a valuable reference for personalized ceftazidime treatment in critically ill children.

## 5 Conclusion

Overall, the PPK model of ceftazidime was successfully established, and individualized dosing regimens for critically ill children were elucidated. It appeared that the body weight and eGFR were the most significant covariates associated with the CL of ceftazidime. The analysis indicated that the current daily dosage for children adequately meets treatment requirements for MIC levels of 2, 4, and 8 mg/L; however, it may not be sufficient for a bacterial infection with an MIC of 16 mg/L, where an elevated dosage regimen may be required. Our research has provided an evidence-based approach for ceftazidime dosage individualization in the critically ill pediatric population. Future research will encompass a broader scope to thoroughly investigate the effects of multidimensional covariates on the PK/PD indicators of ceftazidime.

## Data Availability

The raw data supporting the conclusion of this article will be made available by the authors, without undue reservation.
