# Supplementary material for: Population pharmacokinetics and dose optimization of ceftazidime in critically ill children
Source: Front Pharmacol. 2024 Nov 27;15:1470350. doi: 10.3389/fphar.2024.1470350 (PMC11631598; doi:10.3389/fphar.2024.1470350)
Supplement: Supplementary file 1 [file Table1.DOCX]

Table S1 Hypothesis test results of covariates affecting on ceftazidime clearance (CL).

| **Hypothesis test** | **OFV** | **△OFV** | **P-value** | **Comments** |
| --- | --- | --- | --- | --- |
| Basic model | 630.77 |  |  |  |
| Dose gender affect CL? | 630.78 | 0.01 | >0.05 | NO |
| Dose age affect CL? | 613.55 | -17.22 | <0.05 | YES |
| Dose weight affect CL? | 613.28 | -17.49 | <0.05 | YES |
| Dose hight affect CL? | 615.66 | -15.11 | <0.05 | YES |
| Dose BSA affect CL? | 613.63 | -17.14 | <0.05 | YES |
| Dose BMI affect CL? | 626.25 | -4.52 | <0.05 | YES |
| Dose BUN affect CL? | 630.94 | 0.17 | >0.05 | NO |
| Dose CRE affect CL? | 630.52 | -0.25 | >0.05 | NO |
| Dose UA affect CL? | 629.72 | -1.05 | >0.05 | NO |
| Dose Cys-C affect CL? | 623.49 | -7.28 | <0.05 | YES |
| Dose TBIL affect CL? | 628.93 | -1.84 | >0.05 | NO |
| Dose ALT affect CL? | 629.28 | -1.24 | >0.05 | NO |
| Dose AST affect CL? | 629.28 | -1.49 | >0.05 | NO |
| Dose eGFR affect CL? | 614.97 | -15.80 | <0.05 | YES |

*△OFV, the change of OFV.*

Table S2 PTAs calculated based on the PK model at MIC values (2, 4, 8 and 16 mg/L) for different ceftazidime dosage regimens.

| Patients group | | Dosing regimen | PTA （70% *f*T > MIC） | | | |
| --- | --- | --- | --- | --- | --- | --- |
| Weight  (kg) | eGFR  (mL/min·1.73m^2^) |  | MIC_90_=2 mg/L | MIC_90_=4 mg/L | MIC_90_=8 mg/L | MIC_90_=16 mg/L |
| ＜10 | 30-60 | 50mg/kg q12h | 98.8 | 95.7 | 85.4 | 52.1 |
|  |  | 35mg/kg q12h | 97.7 | 91.9 | 71.1 | 26.7 |
|  |  | 30mg/kg q12h | 96.9 | 88.8 | 63 | 15.4 |
|  |  | 20mg/kg q12h | 93.5 | 77.4 | 35.9 | 2.6 |
|  |  | 17.5mg/kg q12h | 91.9 | 71.1 | 26.7 | 1．1 |
|  |  | 35mg/kg q8h | 100 | 100 | 98.0 | 84.7 |
|  |  | 22.5/kg q8h | 100 | 99.9 | 91.7 | 53.3 |
|  |  | 20mg/kg q8h | 100 | 98.5 | 88.5 | 40.8 |
|  |  | 25mg/kg q6h | 100 | 100 | 99.7 | 92.1 |
|  |  | 20mg/kg q6h | 100 | 100 | 98.9 | 83.2 |
|  |  | 17.5mg/kg q6h | 100 | 100 | 98.2 | 72.5 |
|  |  | 15mg/kg q6h | 100 | 100 | 96.4 | 58.4 |
|  | 60-90 | 50mg/kg q12h | 95.4 | 87.3 | 64.5 | 24.7 |
|  |  | 45mg/kg q12h | 94.4 | 85 | 59.1 | 18.1 |
|  |  | 32.5mg/kg q12h | 91.2 | 75.3 | 41.2 | 6.2 |
|  |  | 30mg/kg q12h | 89.5 | 72.7 | 36.2 | 4.4 |
|  |  | 25mg/kg q12h | 87.3 | 64.5 | 24.7 | 2.1 |
|  |  | 32.5mg/kg q8h | 100 | 98.3 | 90.2 | 55.4 |
|  |  | 25mg/kg q8h | 99.4 | 96.9 | 82.5 | 33.1 |
|  |  | 22.5mg/kg q8h | 99.1 | 95.3 | 77 | 23.8 |
|  |  | 17.5mg/kg q8h | 98.5 | 91.8 | 61.1 | 8.4 |
|  |  | 40mg/kg q6h | 100 | 100 | 99.6 | 94.9 |
|  |  | 35mg/kg q6h | 100 | 100 | 99.2 | 91.8 |
|  |  | 25mg/kg q6h | 100 | 100 | 97.8 | 77.9 |
|  |  | 20mg/kg q6h | 100 | 99.6 | 94.9 | 59.3 |
|  |  | 25mg/kg q8h | 98.3 | 92.4 | 68.3 | 16.7 |
|  | 90-120 | 50mg/kg q12h | 90.0 | 76.0 | 46.8 | 11.6 |
|  |  | 45mg/kg q12h | 88.8 | 73.2 | 41.9 | 8 |
|  |  | 30mg/kg q12h | 81.3 | 55.7 | 19.1 | 1.4 |
|  |  | 30mg/kg q8h | 98.7 | 94.9 | 77.4 | 31.2 |
|  |  | 32.5mg/kg q8h | 98.9 | 95.3 | 81.2 | 36.9 |
|  |  | 25mg/kg q8h | 98.3 | 92.4 | 68.3 | 16.7 |
|  |  | 40mg/kg q6h | 100 | 100 | 98.6 | 88.6 |
|  |  | 30mg/kg q6h | 100 | 99.7 | 96.6 | 74.8 |
|  |  | 22.5mg/kg q6h | 100 | 99.1 | 91.8 | 52.8 |
|  | 120-200 | 75mg/kg q12h | 86.9 | 71.1 | 44.7 | 13.4 |
|  |  | 70mg/kg q12h | 85.5 | 68.5 | 41.7 | 10.9 |
|  |  | 50mg/kg q12h | 78.7 | 56.5 | 25.4 | 4.1 |
|  |  | 30mg/kg q12h | 63.8 | 34.4 | 7.1 | 0.2 |
|  |  | 40mg/kg q8h | 97.6 | 92 | 73.2 | 30.8 |
|  |  | 35/kg q8h | 97.0 | 89.5 | 66.8 | 20.7 |
|  |  | 30mg/kg q8h | 96.2 | 87.1 | 57.1 | 12.1 |
|  |  | 20mg/kg q8h | 92 | 73.2 | 30.8 | 2.2 |
|  |  | 35mg/kg q6h | 100 | 98.9 | 93.7 | 66.3 |
|  |  | 30mg/kg q6h | 100 | 98.5 | 90.5 | 54.5 |
|  |  | 40mg/kg q6h | 100 | 99.1 | 95.2 | 74.5 |
|  |  | 15mg/kg q6h | 98.5 | 90.5 | 54.5 | 4.8 |
| 10-30 | 30-60 | 50mg/kg q12h | 100 | 98.4 | 93.1 | 69.8 |
|  |  | 30mg/kg q12h | 98.9 | 95 | 79.4 | 32.8 |
|  |  | 22.5mg/kg q12h | 98.0 | 90.8 | 63.5 | 12.5 |
|  |  | 15mg/kg q12h | 95 | 79.4 | 32.8 | 1.6 |
|  |  | 12.5mg/kg q12h | 93.1 | 69.8 | 19.9 | 0.4 |
|  |  | 32.5mg/kg q8h | 100 | 100 | 99.1 | 90.3 |
|  |  | 30mg/kg q8h | 100 | 100 | 98.8 | 87.5 |
|  |  | 25mg/kg q8h | 100 | 100 | 97.8 | 79 |
|  |  | 15mg/kg q6h | 100 | 100 | 98.9 | 76 |
|  | 60-90 | 50mg/kg q12h | 98.3 | 93.6 | 80.0 | 43.0 |
|  |  | 40mg/kg q12h | 97.1 | 91.1 | 70.9 | 27.9 |
|  |  | 25mg/kg q12h | 93.6 | 80 | 43 | 5.5 |
|  |  | 20mg/kg q12h | 91.1 | 70.9 | 27.9 | 1.7 |
|  |  | 35mg/kg q8h | 100 | 99.5 | 96.9 | 78.1 |
|  |  | 30mg/kg q8h | 100 | 99.2 | 94.8 | 68 |
|  |  | 25mg/kg q8h | 100 | 98.8 | 91.4 | 54.2 |
|  |  | 27.5mg/kg q6h | 100 | 100 | 99.5 | 91.9 |
|  |  | 25mg/kg q6h | 100 | 100 | 99.2 | 89.2 |
|  |  | 20mg/kg q6h | 100 | 100 | 98.2 | 77 |
|  |  | 55mg/kg q12h | 96.2 | 89 | 70.2 | 31.8 |
|  | 90-120 | 50mg/kg q12h | 95.6 | 87.8 | 65.3 | 25.7 |
|  |  | 30mg/kg q12h | 90.2 | 73.6 | 37.2 | 4.9 |
|  |  | 40mg/kg q8h | 100 | 99 | 94.4 | 70.7 |
|  |  | 32.5mg/kg q8h | 100 | 98.3 | 90.6 | 56.6 |
|  |  | 20mg/kg q8h | 99 | 94.4 | 70.7 | 15.4 |
|  |  | 17.5mg/kg q8h | 98.6 | 92.3 | 62.4 | 8.8 |
|  |  | 32.5mg/kg q6h | 100 | 100 | 99.1 | 90.2 |
|  |  | 30mg/kg q6h | 100 | 100 | 98.8 | 87.6 |
|  |  | 25mg/kg q6h | 100 | 100 | 97.9 | 78.9 |
|  | 120-200 | 60mg/kg q12h | 91.2 | 79 | 52.4 | 16.5 |
|  |  | 50mg/kg q12h | 88.8 | 74.2 | 44.0 | 9.4 |
|  |  | 45mg/kg q12h | 87.8 | 70.2 | 38.8 | 6.8 |
|  |  | 50mg/kg q8h | 99.6 | 97.7 | 90.8 | 69.6 |
|  |  | 40mg/kg q8h | 99 | 96.4 | 86.1 | 50.5 |
|  |  | 30mg/kg q8h | 98.7 | 93.7 | 75.5 | 27.5 |
|  |  | 25mg/kg q8h | 97.7 | 90.8 | 64.8 | 13.8 |
|  |  | 20mg/kg q8h | 96.4 | 86.1 | 50.5 | 5.7 |
|  |  | 12.5mg/kg q8h | 90.8 | 64.8 | 13.8 | 0.2 |
|  |  | 40mg/kg q6h | 100 | 100 | 98.4 | 87.1 |
|  |  | 30mg/kg q6h | 100 | 99.4 | 95.7 | 71.5 |
|  |  | 22.5mg/kg q6h | 100 | 98.8 | 90.9 | 48.5 |
| 30-50 | 30-60 | 50mg/kg q12h | 100 | 99.0 | 95.0 | 77.6 |
|  |  | 40mg/kg q12h | 100 | 98.5 | 92.3 | 65.5 |
|  |  | 30mg/kg q12h | 99.4 | 96.9 | 85.3 | 42.6 |
|  |  | 25mg/kg q12h | 99 | 95.0 | 77.6 | 28.1 |
|  |  | 20mg/kg q12h | 99 | 95.0 | 77.6 | 28.1 |
|  |  | 12.5mg/kg q12h | 95 | 77.6 | 28.1 | 0.7 |
|  |  | 10mg/kg q12h | 92.3 | 63.5 | 11.9 | 0.2 |
|  |  | 30mg/kg q8h | 100 | 100 | 99.3 | 91.4 |
|  |  | 20mg/kg q8h | 100 | 100 | 97.1 | 69.5 |
|  |  | 22.5mg/kg q8h | 100 | 100 | 98.2 | 79.2 |
|  |  | 15mg/kg q6h | 100 | 100 | 99.4 | 84.0 |
|  |  | 13mg/kg q6h | 100 | 100 | 98.9 | 71.6 |
|  |  | 12.5mg/kg q6h | 100 | 100 | 98.5 | 67.0 |
|  |  | 50mg/kg q12h | 98.8 | 96.0 | 85.9 | 53.5 |
|  | 60-90 | 35mg/kg q12h | 97.7 | 92.2 | 72.0 | 27.6 |
|  |  | 32.5mg/kg q12h | 97.5 | 91.1 | 69.2 | 22.2 |
|  |  | 30mg/kg q12h | 97 | 89.3 | 64.4 | 16.5 |
|  |  | 17.5mg/kg q12h | 92.2 | 72 | 27.6 | 1.3 |
|  |  | 15mg/kg q12h | 89.3 | 64.4 | 16.5 | 0.4 |
|  |  | 32.5mg/kg q8h | 100 | 100 | 99.7 | 92.7 |
|  |  | 30mg/kg q8h | 100 | 99.8 | 96.9 | 75.8 |
|  |  | 22.5mg/kg q8h | 100 | 99.1 | 92.2 | 54.4 |
|  |  | 25mg/kg q6h | 100 | 100 | 99.7 | 92.7 |
|  |  | 17.5mg/kg q6h | 100 | 100 | 98.2 | 73.7 |
|  |  | 50mg/kg q12h | 96.9 | 90.4 | 71.4 | 31.7 |
|  | 90-120 | 47.5mg/kg q12h | 97.0 | 91.5 | 74.1 | 35.0 |
|  |  | 45mg/kg q12h | 96.2 | 89.4 | 69.3 | 28.2 |
|  |  | 40mg/kg q12h | 96.1 | 87.7 | 63.3 | 20.0 |
|  |  | 30mg/kg q12h | 93.5 | 80.1 | 47.1 | 7.4 |
|  |  | 27.5mg/kg q12h | 92.7 | 77.5 | 41.3 | 5.4 |
|  |  | 25mg/kg q12h | 91.5 | 74.1 | 35.0 | 3.3 |
|  |  | 35mg/kg q8h | 100 | 99.1 | 94.9 | 70.4 |
|  |  | 30mg/kg q8h | 100 | 98.8 | 92.2 | 59.5 |
|  |  | 30mg/kg q6h | 100 | 100 | 99.3 | 91.4 |
|  |  | 25mg/kg q6h | 100 | 100 | 98.8 | 85.5 |
|  |  | 22.5mg/kg q6h | 100 | 100 | 98.2 | 79.2 |
|  |  | 20mg/kg q6h | 100 | 100 | 97.1 | 68.9 |
|  | 120-200 | 60mg/kg q12h | 93.9 | 85 | 62.1 | 24.6 |
|  |  | 42.5mg/kg q12h | 90.3 | 75.3 | 45.4 | 8.9 |
|  |  | 40mg/kg q12h | 89.3 | 74.0 | 41.7 | 7.4 |
|  |  | 37.5mg/kg q12h | 88.4 | 71.4 | 38.2 | 6.0 |
|  |  | 35mg/kg q12h | 87.8 | 68.7 | 33.8 | 4.5 |
|  |  | 35mg/kg q8h | 99.4 | 97 | 87.0 | 50.4 |
|  |  | 32.5mg/kg q8h | 99.2 | 96.8 | 85.2 | 42.8 |
|  |  | 27.5mg/kg q8h | 98.9 | 95 | 78.1 | 29.8 |
|  |  | 25mg/kg q8h | 98.7 | 94 | 72.8 | 21.6 |
|  |  | 20mg/kg q8h | 97.9 | 90.2 | 58.4 | 8.8 |
|  |  | 40mg/kg q6h | 100 | 100 | 99.1 | 91.3 |
|  |  | 27.5mg/kg q6h | 100 | 100 | 96.9 | 73.2 |
|  |  | 25mg/kg q6h | 100 | 99.5 | 95.3 | 66.9 |
|  |  | 20mg/kg q6h | 100 | 99.1 | 91.3 | 47.3 |
| 50-70 | 30-60 | 50mg/kg q12h | 100 | 99.3 | 96.3 | 82.0 |
|  |  | 47.5mg/kg q12h | 100 | 99.1 | 95.6 | 79.5 |
|  |  | 45mg/kg q12h | 100 | 99.0 | 95.1 | 76.7 |
|  |  | 35mg/kg q12h | 100 | 98.5 | 91.3 | 60.9 |
|  |  | 20.5mg/kg q12h | 98.9 | 94.2 | 70.7 | 17.0 |
|  |  | 20mg/kg q12h | 98.8 | 93.7 | 69.8 | 14.6 |
|  |  | 17.5mg/kg q12h | 98.5 | 91.3 | 60.9 | 8.7 |
|  |  | 12.5mg/kg q12h | 96.3 | 82 | 33.0 | 1.3 |
|  |  | 10.5mg/kg q12h | 94.6 | 71.9 | 18.3 | 0.3 |
|  |  | 10mg/kg q12h | 93.7 | 69.8 | 14.6 | 0.2 |
|  |  | 7.5mg/kg q12h | 88.0 | 49.7 | 3.6 | 0.1 |
|  |  | 27.5mg/kg q8h | 100 | 100 | 98.0 | 74.4 |
|  |  | 20mg/kg q8h | 100 | 100 | 98.0 | 74.4 |
|  |  | 15mg/kg q8h | 100 | 99.7 | 93.3 | 48.3 |
|  |  | 12.5mg/kg q6h | 100 | 100 | 99.3 | 90.8 |
|  |  | 50mg/kg q12h | 99.0 | 97.0 | 88.2 | 58.3 |
|  | 60-90 | 32.5mg/kg q12h | 98.3 | 93.2 | 73.4 | 27.2 |
|  |  | 30mg/kg q12h | 97.7 | 91.8 | 69.8 | 21.3 |
|  |  | 20mg/kg q12h | 95 | 82.3 | 42.7 | 3.9 |
|  |  | 17.5mg/kg q12h | 93.8 | 77 | 32.8 | 2.3 |
|  |  | 15mg/kg q12h | 91.8 | 69.8 | 21.3 | 0.7 |
|  |  | 12.5mg/kg q12h | 88.2 | 58.3 | 11.0 | 0.2 |
|  |  | 32.5mg/kg q8h | 100 | 100 | 98.3 | 85.3 |
|  |  | 30mg/kg q8h | 100 | 100 | 97.5 | 80.9 |
|  |  | 27.5mg/kg q8h | 100 | 100 | 97.0 | 74.5 |
|  |  | 25mg/kg q8h | 100 | 99.5 | 95.3 | 68.0 |
|  |  | 20mg/kg q8h | 100 | 100 | 98.3 | 85.3 |
|  |  | 22.5mg/kg q6h | 100 | 100 | 99.7 | 71.1 |
|  |  | 20mg/kg q6h | 100 | 100 | 99.3 | 87.4 |
|  | 90-120 | 45mg/kg q12h | 97.3 | 91.7 | 74.0 | 33.4 |
|  |  | 25mg/kg q12h | 93.2 | 77.9 | 40.4 | 4.7 |
|  |  | 22.5mg/kg q12h | 91.7 | 74.0 | 33.4 | 2.6 |
|  |  | 20mg/kg q12h | 89.5 | 68.6 | 25.2 | 1.2 |
|  |  | 35mg/kg q8h | 100 | 99.4 | 95.8 | 75.6 |
|  |  | 25mg/kg q8h | 100 | 98.6 | 90.4 | 50.7 |
|  |  | 17.5mg/kg q8h | 99.4 | 95.8 | 75.6 | 17.4 |
|  |  | 27.5mg/kg q6h | 100 | 100 | 99.4 | 90.8 |
|  |  | 25mg/kg q6h | 100 | 100 | 99.4 | 90.8 |
|  |  | 50mg/kg q12h | 93.8 | 83.7 | 58.7 | 19.0 |
|  | 120-200 | 40mg/kg q12h | 91.6 | 77.8 | 46.6 | 9.1 |
|  |  | 35mg/kg q12h | 89.5 | 73.6 | 39.2 | 6.2 |
|  |  | 30mg/kg q12h | 87.8 | 66.9 | 29.9 | 3.0 |
|  |  | 32.5mg/kg q8h | 99.6 | 97.3 | 87.6 | 49.9 |
|  |  | 25mg/kg q8h | 99 | 95.1 | 77.4 | 27.2 |
|  |  | 20mg/kg q8h | 98.5 | 92.4 | 65.1 | 11.3 |
|  |  | 17.5mg/kg q8h | 97.9 | 89.5 | 55.0 | 6.0 |
|  |  | 40mg/kg q6h | 100 | 100 | 99.3 | 93.1 |
|  |  | 37.5mg/kg q6h | 100 | 100 | 99.1 | 91.6 |
|  |  | 25mg/kg q6h | 100 | 100 | 96.7 | 71.3 |
|  |  | 20mg/kg q6h | 100 | 99.3 | 93.1 | 53.6 |
|  |  | 17.5mg/kg q6h | 100 | 99.0 | 89.5 | 38.1 |
